# Upregulated long intergenic non-protein coding RNA 1094 (LINC01094) is linked to poor prognosis and alteration of cell function in colorectal cancer

**DOI:** 10.1080/21655979.2022.2051839

**Published:** 2022-03-24

**Authors:** Guangliang Zhang, Yingjie Gao, Zhen Yu, Hui Su

**Affiliations:** a Oncology Department, Liaocheng People’s Hospital, Liaocheng, China; b Intervention Therapy Department, Liaocheng People’s Hospital, Liaocheng, China

**Keywords:** Colorectal cancer, LINC01094, prognosis, growth

## Abstract

Colorectal cancer (CRC) showed high cancer-related mortality in recent years partly due to the absence of an effective prognostic predictor. This research intended to evaluate the prognostic value and potential role of long intergenic non-protein coding RNA 1094 (LINC01094) in CRC. In this work, we evaluated the LINC01094 level in 122 CRC patients’ tissues and in human CRC cell lines. We explored the ability of LINC01094 in overall survival and progression-free survival estimate. The effect of LINC01094 dysregulation on the CRC cells was investigated. LINC01094 is highly expressed in CRC tissues and cells than normal ones. This high expression was correlated with absent vascular invasion, positive lymph node metastasis, and advanced TNM stage. With the result of Kaplan-Meier analysis and multivariate Cox’s proportional hazard analysis, LINC01094 was an effective biomarker for CRC overall survival. Downregulation of LINC01094 impeded the malignant biological behavior (proliferation, invasion, and migration) of CRC cells, while overexpression of LINC01094 boosted that maybe by sponging miR-1266-5p. LINC01094 might function as an oncogene in CRC and allowed the discovery of a new biomarker for prognosis and therapy of CRC.

## Graphical Abstract

**Figure:**
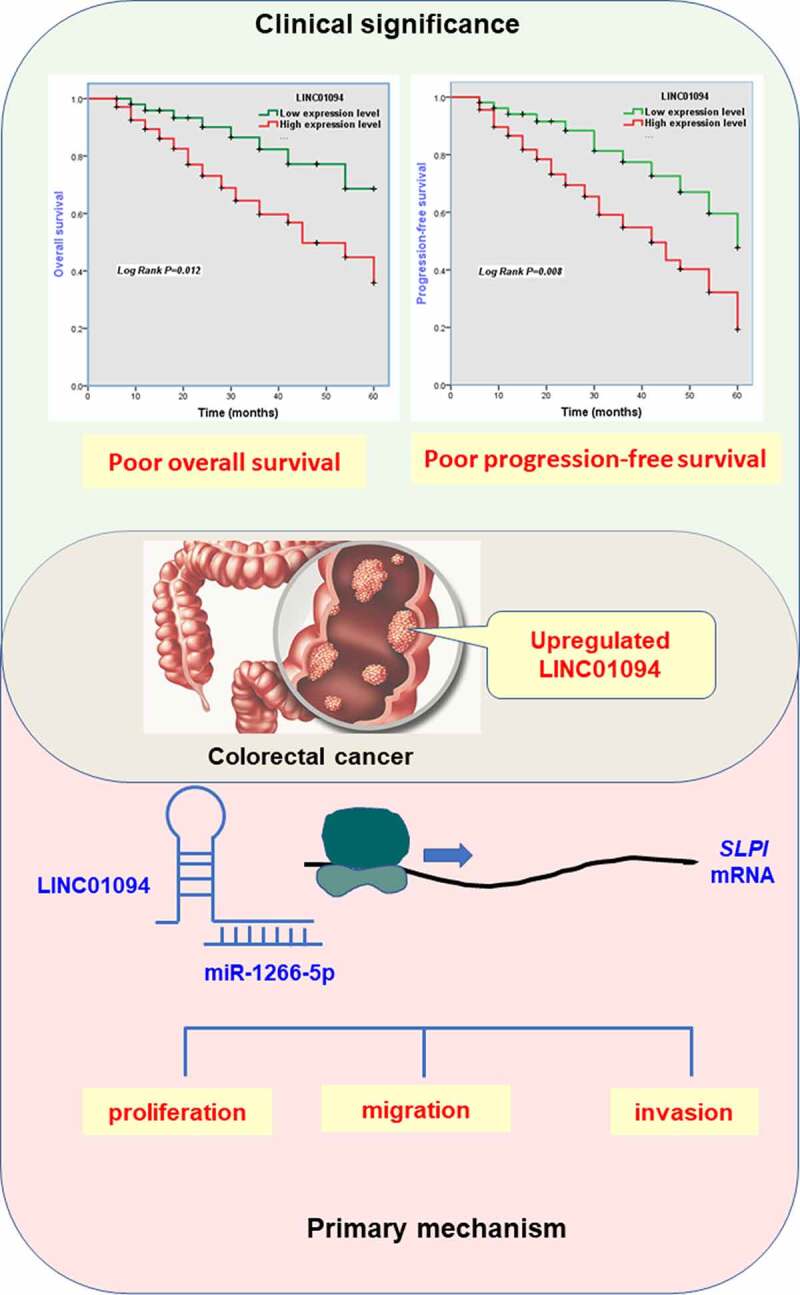

## Introduction

With approximately 2 million incidences in 2020, colorectal cancer (CRC) came in fourth in all types of cancers worldwide [1]. The estimated number of deaths in 2020 due to CRC ranked second with a mortality of 935,173 [2,3]. What is more serious is the incidence rates for carcinoma of the colon and rectum will be increased drastically in 2030 based on the current data [4]. CRC is still a huge cancer burden in China as the third most diagnosed and the fifth lethal cancer [5]. The 5-year survival relative rate for CRC ranges from 90% to 14% due to the stage at diagnosis [6]. The outcome of patients with CRC largely depends on the essential and additional prognostic factors apart from the tumor-node-metastasis (TNM) staging system [7]. Moreover, the development of novel and promising biomarkers for CRC prognosis would be an important avenue for the prediction of CRC patients’ survival and contribute to the formulation of treatment plans therapy strategies [8,9].

Long non-coding RNAs (lncRNAs), a series of non-coding genes, have been known as the gene expression regulators at the transcription level [10]. They act either as molecular scaffolds (mediating chromatin remodelers targeted to specific genomic regions) or decoys (sequestering chromatin remodelers or transcription factors from their action site) to regulate gene expression in *cis* or *trans* [11]. LncRNAs are becoming novel indispensable participants involved in cancer development and progression [10]. Indeed, they are related to the risk for many cancer types including CRC [12–14]. Additionally, lncRNAs are commonly expressed in disease- or tissue-specific manners, while the dysregulation of a certain lncRNA is correlated with tumor type, the stage, and prognosis [15]. Thus, all these offer lncRNAs the potential as biomarkers and the therapeutic targets for cancers including CRC [16]. Long intergenic non-protein coding RNA 01094 (LINC01094) is involved in the progression of clear cell renal cell carcinoma, ovarian cancer, and glioma [17–19]. Though dysregulation of LINC01094 in CRC has been screened [20], its role in CRC hasn’t been revealed.

Here, we hypothesize that LINC01094 might play a role in CRC and may serve as a novel prognostic biomarker. In this work, we intended to evaluate the expression of LINC01094 in CRC and explore whether it is involved in the progression of CRC. Apart from this, we also included the predictive power of LINC01094 both for overall survival and progression-free survival.

## Material and methods

### Patients and specimens

Patients who were diagnosed as CRC according to the Chinese Society of Clinical Oncology diagnosis and treatment guidelines for colorectal cancer 2010 version and undergone surgical resection at Liaocheng People’s Hospital were screened. Those with enough samples for the experiment and complete medical records related to our study were taken into consideration. In these patients, the ones who received any treatment related to cancers or had a history of other cancers were eliminated. Finally, 122 cases between January 2011 and August 2015 were enrolled and their tissues were collected, containing paired cancerous tissues and adjacent normal tissues. The period from CRC diagnosis until disease progression or death was defined as progression-free survival. Overall survival was defined as the period between the date of CRC diagnosis and the date of death or five-year follow-up ends.

Written formed consent was obtained from all participants clear with the usage of tissues for this research. The Institutional Review Board of the Liaocheng People’s Hospital (No. 2011006) approved this study.

### Cell’s culture

FHC (human large intestine and colon normal epithelial cell), SW480 (human colorectal adenocarcinoma; Dukes’ type B), SW620 (human colorectal adenocarcinoma; Dukes’ type C), LoVo (human colorectal adenocarcinoma; Dukes’ type C, grade IV), HCT116 (human colorectal carcinoma), and SW1463 cells (human colorectal adenocarcinoma; Dukes’ type C) were purchased and utilized (ATCC, USA). SW480, SW620, and SW1463 cells were cultured in L-15 Medium (Gibco, USA) supplemented with 10% fetal bovine serum (FBS, Sigma-Aldrich, USA) at an atmosphere of 100% air. FHC, LoVo, and HCT-116 cells were cultured in DMEM (Gibo, USA) supplemented with 10% FBS (Sigma-Aldrich, USA) and 1% penicillin and streptomycin (Sigma-Aldrich, USA) in a humidified incubator containing 5% CO_2_ and 95% air.

### Cell’s transfection

SW620 and LoVo cells were seeded in six-well plates for 24 to achieve about 80% of the cell confluence. Then the LINC01094 overexpression vector (oe-LINC01094), the control overexpression vector (oe-NC), the small interfering RNA (siRNA) against LINC01094 (si-LINC01094), or the control siRNA (si-NC) were transfected into cells with the help of JetPrime transfection reagent (Polyplus, USA) following the manufacturer’s protocol [21]. miR-1266-5p mimic (miR-1266-5p), mimic negative control (miR-con), miR-1266-5p inhibitor (anti-miR-1266-5p) and the inhibitor negative control (anti-miR-con) were transfected into cells using Lipofectamine RNAiMax (Invitrogen, USA) [22]. All plasmids, vectors and sequences were delegated to the GenePharm (Shanghai, China).

### RNA extraction

For extraction of RNA from CRC tissues, samples were subjected to homogenization in an Omni Bead Ruptor12 Homogenizer (Cole-Parmer, USA) in the presence of Trizol (Invitrogen, USA). RNA from cells was extracted using Trizol (Invitrogen) as per the manufacturer’s indication [23].

### Nuclear/Cytoplasmic Extraction

Nuclear and cytoplasmic RNA were extracted from the SW620 and LoVo cells using the PARIS™ Kit (Invitrogen, USA) as described previously [24]. For the subcellular location of LINC01094, U6 and glyceraldehyde 3-phosphate dehydrogenase (GAPDH) were used as the nuclear and cytoplasmic index, respectively, in reverse transcription-quantitative PCR (RT-qPCR).

### RNA pull-down assay

RNA pull-down assay was conducted based on the established instruction of Pierce™ Magnetic RNA-Protein Pull-Down Kit (Thermo Scientific, USA) [25]. The cell lysates of SW620 were prepared to mix with the biotinylated probes for LINC01094 overnight and followed by incubation with magnetic beads. The RNA-RNA binding mixture washed finally was analyzed by RT-qPCR.

### RT-qPCR detection

Complementary DNA was produced by High-Capacity cDNA Reverse Transcription Kit (Appliedbiosystems, USA). RT-qPCR for LINC01094 was performed using Maxima SYBR Green/ROX qPCR Master Mix (Thermo Scientific, USA). LINC01094 level in samples were normalized using GADPH as an endogenous control. MiR-1266-5p expression was quantified using TaqMan microRNA assays along with TaqMan Universal PCR Master Mix (Appliedbiosystems, USA) per the manufacturer’s protocol, and normalized to U6. And the relative levels of RNAs were calculated as the 2^−ΔΔCt^ methods. The primer sequences were listed in Supplementary Table 1.

### MTT assay for cell proliferation

Cell proliferation was evaluated via MTT Assay Kit (ab211091, Abcam) that is based on 3-(4,5-dimethylthiazol-2-yl)-2,5-diphenyltetrazolium bromide as per manufacturer’s instruction [26]. Put simply, transfected SW620 and LoVo Cells were seeded in 96 well plates and incubated. Then the serum-free media mixed with MTT reagent was used to replace serum-containing media in the cell cultures every 24 hours within 72 hours, that is, 0 h, 24, 48, and 72 hours, following another incubation for 3 h at 37°C. The absorbance was analyzed with SpectraMax iD3 microplate reader. Each sample was seeded by triplet and three assays were performed.

### Transwell migration and Matrigel invasion assays

Costar Transwell inserts (Corning, USA) and Matrigel Invasion Chambers (BD, USA) were purchased to evaluate the cell migration and invasion respectively [27]. For migration assay of SW620 cells, transfected cells were harvested and seeded (1× 10^4^ cells per well) onto transwell membranes in serum-free L-15 Medium. Lower chambers of companion plates were filled with 10% FBS L-15 Medium as a chemoattractant. After 24 hours incubation at 37°C, non-migrated cells on the inner surfaces were scrapped with PBS-soaked cotton, while migrated cells were fixed, washed and stained. Five fields were imaged from each transwell membrane using the Live Cell Imaging System (Olympus, Japan), and the number of migrated cells was manually counted. For migration assay of LoVo cells, except for 2 × 10^5^ cells per well and 20 hours of incubation, the rest of the steps are the same as the migration assay of SW620 cells. As for the invasion assays, the test steps are basically the same as above, except for 36 hours of incubation for SW620 cells and 48 hours of incubation for LoVo cells respectively.

### Bioinformatic analysis

To predict the target miRNAs of LINC01094, a comprehensive resource of single nucleotide polymorphisms in human lncRNAs, lncRNASNP2 (http://bioinfo.life.hust.edu.cn/lncRNASNP#!/) was retrieved. Then, TargetScan (http://www.targetscan.org/vert_72/) was searched and predicted the biological targets of miRNA (miR-1266-5p).

### Dual-luciferase reporter assay

The fragments of LINC01094 that contain the wild-type or mutated type of miR-1266-5p pairing sequences were provided by GenePharma (Shanghai, China). The sequence of wide-type LINC01094 or *SLPI* 3’-untranslated region (3’-UTR) was cloned into pGL3 luciferase reporter vector to form LINC01094-WT or *SLPI*-WT. The sequence of mutated-type LINC01094 or *SLPI* 3’-UTR was cloned into pGL3 luciferase reporter vector to obtained LINC01094-MUT or *SLPI*-MUT. SW620 cells were cultured in 24 well plates and co-transfected with the reporter plasmid and the miR-1266-5p mimics or inhibitors. 48 hours later, the relative luciferase activity was measured.

### Statistical analysis

Analysis of data statistics was performed using GraphPad Prism 7 and IBM SPSS statistics 22. Differences between groups were estimated by the *t-*test, one-way analysis of variance, or two-way analysis of variance when appropriate. Chi-square tests were performed to evaluate the correlations between LINC01094 level and clinical factors. Survival estimates were performed by Kaplan-Meier analysis and compared with the log-rank test for time-to-event analysis. Multi-multivariate Cox’s proportional hazard analysis was used to estimate the risk ratio of each parameter for death. *P* values less than 0.05 were considered statistically significant.

## Results

Previous studies demonstrated that LINC01094 is dysregulated in CRC [20]. However, its clinical significance, effect on CRC progression, and the molecular mechanism underlying remain unexplored. Here, we first confirmed the expression pattern of LINC01094 in CRC. Then, the prognostic value of LINC01094 was assessed. In addition, the role of LINC01094 in regulating the biological behavior of CRC cells was explored. After predicting and confirming the downstream miRNA/mRNA axis of LINC01094, we found that the oncogenic role of LINC01094 was dependent on its regulation of the miR-1266-5p/SLPI axis.

### Levels of LINC01094 increase in tissues of CRC patients and CRC cells

**Figure 1.**
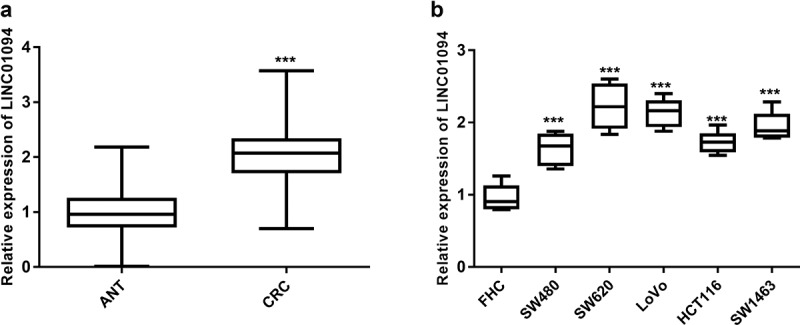
LINC01094 level was upregulated in colorectal cancer tissues and cells. (a). Box plots of the LINC01094 expression levels in adjacent normal tissue (ANT) and colorectal cancer tissues (CRC) by RT-qPCR. (b). Boxplots of the LINC01094 expression levels in colon normal epithelial cell FHC and colorectal cancer cells, SW480, SW620, LoVo, HCT116, and SW1463 by RT-qPCR. ***P < 0.001.

A total of 122 CRC samples and a series of CRC and normal cells were subjected to RT-qPCR to determine the level of LINC01094. The tumor tissue samples evaluated had significantly elevated expression of LINC01094 compared with ANT (*P* < 0.001, Figure 1a). Likewise, the analysis of LINC01094 in CRC cells (SW480, SW620, LoVo, HCT116, and SW1463 cells) highlighted the upregulated expression of LINC01094 (*P*< 0.001, Figure 1b) compared to normal colon cells FHC. Given the outstanding LINC01094 expression levels, SW620 and/or LoVo were used for subsequent experiments.

### Association between the LINC01094 level and clinicopathological factors in CRC patients

Next, we analyzed the association between the LINC01094 level and the clinicopathological data of 122 CRC patients. The CRC patients’ clinicopathological factors are summarized in Table 1. The analysis was carried out by dividing the CRC patients into two groups using the median relative level of LINC01094, 2.057, in all CRC patients as a cutoff. As shown in Table 1, a high LINC01094 level tended to correlate with the absence of vascular invasion (*P* = 0.012) and was remarkably associated with positive lymph node metastasis (*P* = 0.003) and advanced TNM stage (*P* = 0.007).

**Table 1.** Key characteristics and their association with clinical LINC01094 expression in patients with colorectal cancer

Characteristics	Cases (n = 122)	LINC01094 expression		*P*
		Low (n = 54)	High (n = 68)	
Age				
≤ 60	61	24	37	0.274
> 60	61	30	31	
Gender				
Female	59	23	36	0.256
Male	63	31	32	
Differentiation				
Well/Moderate	68	33	35	0.287
Poor	54	21	33	
Vascular invasion				
Absent	93	47	46	0.012
Present	29	7	22	
Lymph node metastasis				
Negative	85	45	40	0.003
Positive	37	9	28	
TNM stage				
I–II	79	42	37	0.007
III–IV	43	12	31	

### Correlation of LINC01094 expression with prognosis

**Figure 2.**
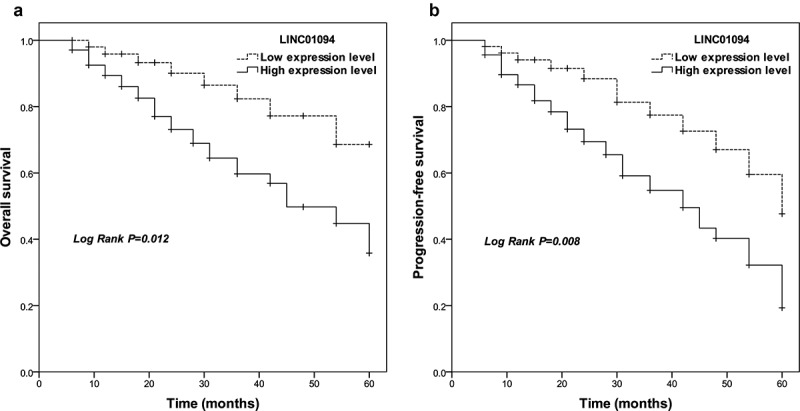
Survival estimates were calculated by Kaplan-Meier analysis and compared with the log-rank test for LINC01094 in colorectal cancer. (a). Patients with high LINC01094 expression had a shorter overall survival time than those with low LINC01094 levels. Log-rank test *P* = 0.012. (b). Patients with high LINC01094 expression represented a shorter progression-free survival time than those with low LINC01094 levels. Log-rank test *P* = 0.008.

Given the result above, LINC01094 was analyzed to investigate its association with the clinical overall outcome of CRC patients. In Kaplan-Meier statistics using the log-rank test, CRC patients with high LINC01094 expression levels had significantly lower overall survivals (*P* = 0.012) compared to those with low LINC01094 expression levels (Figure 2a). A significant difference was also observed for progression-free survival among patients with high and low LINC01094 (*P* = 0.008; Figure 2b). After evaluation with multi-multivariate Cox’s analysis, LINC01094 level was an independent prognostic factor (Risk ratio: 4.361, 95%CI: 1.822–10.438, *P*= 0.001, Table 2)

**Table 2.** Multivariate analyses of risk factors for cumulative overall survival in colorectal cancer patients

Characteristics	Multivariate analysis		
	Risk ratio	95% CI	*P*
LINC01094 (High to Low)	4.361	1.822–10.438	0.001
Age (> 60 to ≤ 60)	1.437	0.670–3.083	0.352
Gender (Male to Femal)	1.481	0.669–3.282	0.333
Differentiation (Poor to Well/Moderate)	1.344	0.638–2.828	0.436
Vascular invasion (Present to Absent)	1.987	0.776–5.090	0.152
Lymph node metastasis (Positive to Negative)	3.729	1.188–11.702	0.024
TNM stage (III–IV to I–II)	3.001	1.118–8.052	0.029

### Downregulation of LINC01094 impeded the malignant biological behavior of CRC cells

**Figure 3.**
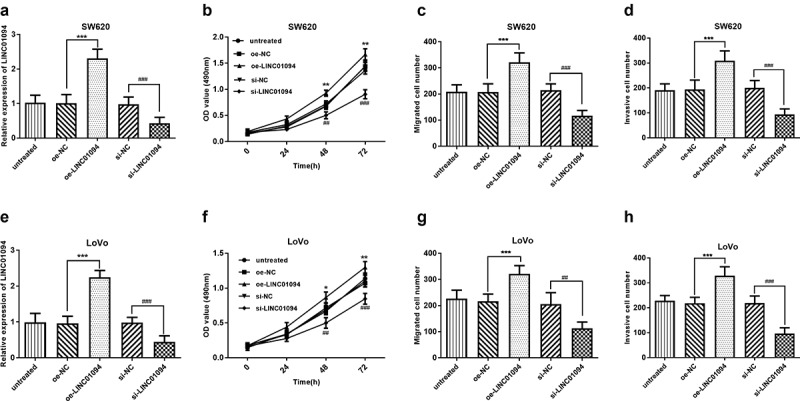
Downregulation of LINC01094 impeded the malignant biological behavior of CRC cells while overexpression hastened that. (a). Overexpression of LINC01094 was achieved by transfection of oe-LINC01094, and downregulation of LINC01094 by transfection of si-LINC01094 in SW620 cells. (b-d). Overexpression of LINC01094 promoted cell proliferation, migration and invasion, while downregulation of LINC01094 impeded the biological behavior of SW620 cells compared with the respective negative control. (e). Overexpression of LINC01094 was achieved by transfection of oe-LINC01094, and downregulation of LINC01094 by transfection of si-LINC01094 in LoVo cells. (f-h). Overexpression of LINC01094 promoted cell proliferation, migration, and invasion, while downregulation of LINC01094 impeded those biological behaviors of LoVo cells compared with the respective negative control. *P < 0.05, **P < 0.01, ***P < 0.001 (oe-LINC01094 vs. oe-NC). ^##^*P* < 0.01, ^###^*P* < 0.001 (si-LINC01094 vs. si-NC).

To investigate whether LINC01094 was involved in CRC cell malignant biological behavior, we inhibited LINC01094 using small interfering RNA in the cell lines with high expression of LINC01094, SW620 and LoVo (*P* < 0.001, Figure 3a **and 3E**) and then carried out MTT assay and Transwell migration and Matrigel invasion assays. Our results showed that inhibition of LINC01094 significantly restricted SW620 cell proliferation in MTT assay while overexpression of LINC01094 increased that (*P* < 0.01, Figure 3b). The Transwell analysis revealed the migration was significantly suppressed in SW620 cell line after LINC01094 siRNA transfection compared with oe-NC transfection, but hoisted by overexpression of LINC01094 (*P* < 0.001, Figure 3c). The Matrigel invasion assays found that inhibition of LINC01094 decreased the cell invasion, nevertheless, upregulation of LINC01094 promoted it (*P* < 0.001, Figure 3d). These results were confirmed using another cell line, LoVo, and the results like those in the SW620 cell line were observed (*P* < 0.05, Figure 3e-h).

### LINC01094 is a sponge of miR-1266-5p

**Figure 4.**
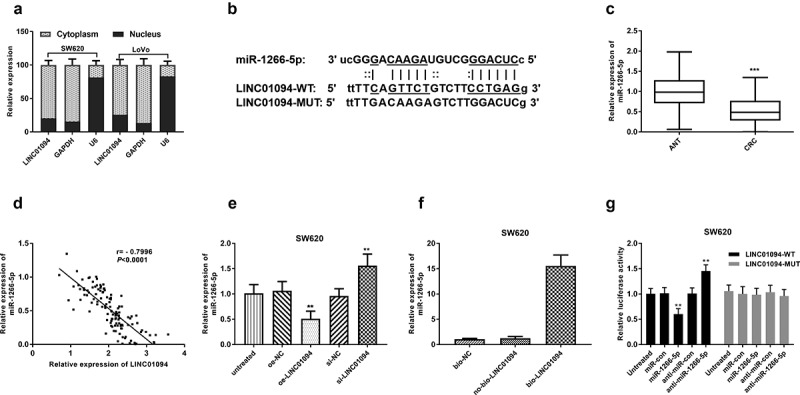
LINC01094 is a sponge of miR-1266-5p. (a). LINC01094 was mainly a cytoplasmic-localized lncRNA by the nuclear/cytoplasmic extraction assay. (b). Prediction of binding sequences involving LINC01094 3’-UTR and miR-1266-5p. (c). Boxplots of the miR-1266-5p expression levels in adjacent normal tissue (ANT) and colorectal cancer tissues (CRC) by RT-qPCR. (d). Correlation between the LINC01094 and miR-1266-5p expression levels analyzed by Pearson r analysis. (e). Overexpression of LINC01094 caused a reduction of miR-1266-5p expression level in SW620 cells, whereas knockdown of LINC01094 increased the level of miR-1266-5p. (f). Detection of relative expression of miRNAs by RT-qPCR in RNA pull-down assay by different biotinylated RNAs. (f). The results of dual-luciferase reporter assay in SW620 cells. **P < 0.01.

The investigation of lncRNAs has expanded to sponges of potential miRNAs, also recognized as competing endogenous RNAs (ceRNAs) [28]. With the nuclear/cytoplasmic extraction assay, we found LINC01094 was a mainly cytoplasmic-localized lncRNA (Figure 4a). To screen the putative miRNAs interacting with LINC01094, the online bioinformatics tool (lncRNASNP2) was utilized. miR-1266-5p was found to possibly bind to LINC01094 (Figure 4b). The miR-1266-5p expression levels in CRC tissues were significantly higher than those in adjacent normal tissues by RT-qPCR (*P* < 0.001, Figure 4c). The LINC01094 expression levels negatively correlated with the miR-1266-5p expression levels in CRC tissues analyzed by Pearson r analysis (*P* < 0.001, Figure 4d). RT-qPCR showed that LINC01094 siRNA led to an increase of miR-1266-5p expression in SW620 cells, whereas LINC01094 overexpression brought about a reduction of miR-1266-5p (*P* < 0.01, Figure 4e). To further confirm the interaction between LINC01094 and miR-1266-5p, we performed RNA pull-down assay. A higher level of miR-1266-5p was observed in bio-LINC01094 pellet than those in no-bio-LINC01094 (*P* < 0.001, figure 4f). Moreover, the luciferase activity of wild-type LINC01094 was decreased by miR-1266-5p mimics and increased by miR-1266-5p inhibitor, while miR-1266-5p mimics or miR-12566-5p inhibitor failed to influence the luciferase activity of the LINC01094-MUT group (*P* < 0.01, Figure 4g). All these results suggest that LINC01094 interacted with miR-1266-5p.

### LINC01094 sponges miR-1266-5p to promote proliferation, migration, and invasion in CRC cells

**Figure 5.**
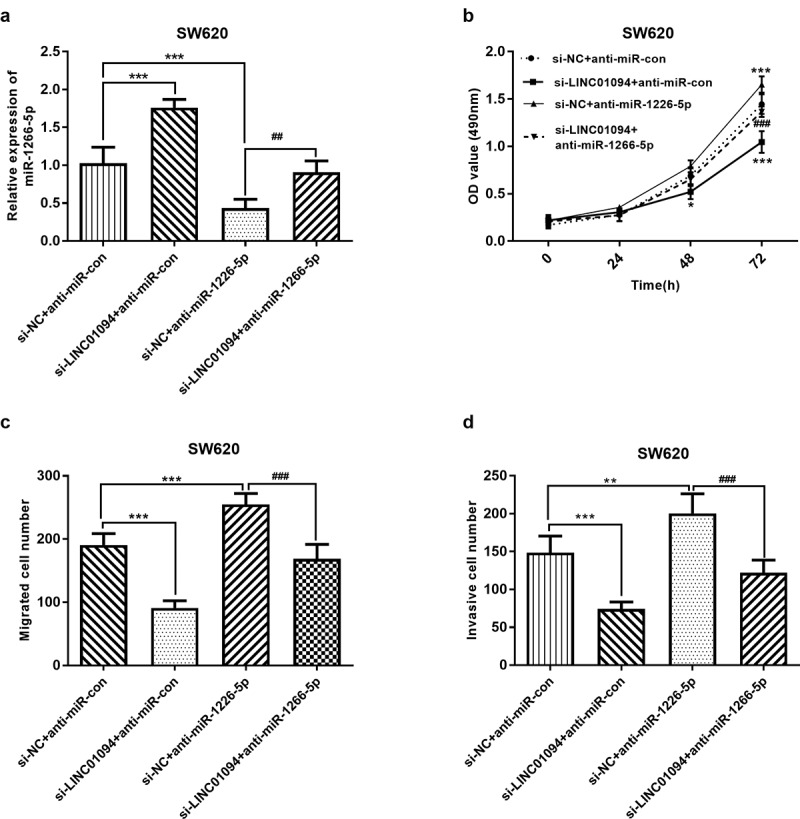
Dynamic effects of LINC01094 and its target miR-1266-5p on CRC cell proliferation, migration and invasion. SW620 cells were co-transfected with si-LINC01094 and anti-miR-1266-5p. (a). Transfection efficiency was detected by RT-qPCR. (b). Cell viability was examined by MTT assay. (c). Cell migratory capacity was examined by Costar Transwell inserts (Corning, USA). (d). Cell invasive capacity was examined by Matrigel Invasion Chambers. *P < 0.05, **P < 0.01, ***P < 0.001, compared to the si-NC+anti-miR-con group; ##*P* < 0.01, ###*P* < 0.001, compared to the si-NC+anti-miR-1266-5p group.

Subsequently, we explored whether LINC01094 promotes the malignant behavior of CRC cells via miR-1266-5p. Given the upregulation of LINC01094 in CRC, LINC01094 si-RNA and miR-1266-5p inhibitor were used to moderate the expression of miR-1266-5p (*P* < 0.01, Figure 5a). MTT assay and transwell assays were performed to investigate the effects of LINC01094 and miR-1266-5p on the proliferation, migration, and invasion of CRC cells in vitro. The decrease in the proliferation of CRC cells after knockdown of LINC01094 was markedly abrogated by silencing miR-1266-5p expression (*P* < 0.05, Figure 5b). Moreover, the decrease in the number of migrated and invaded CRC cells upon knockdown of LINC01094 was markedly weakened by silencing miR-1266-5p (*P* < 0.01, Figure 5c-d). Accordingly, LINC01094 enhanced the proliferative, migratory, and invasive abilities of CRC cells via miR-1266-5p.

### miR-1266-5p targets secretory leukocyte protease inhibitor (SLPI) in CRC

**Figure 6.**
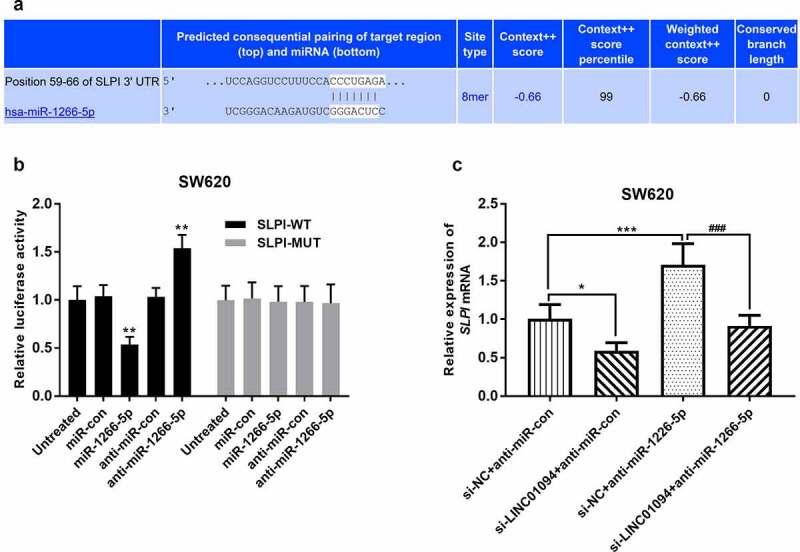
miR-1266-5p binds to the *SLPI* 3’-UTR. (a). The potential binding sites of miR-1266-5p in *SLPI* 3’-UTR. (b). Luciferase reporter assays were performed by constructing luciferase reporter vectors to validate the predicted binding of miR-590-3p to the *SLPI* 3’-UTR. **P < 0.01, ***P < 0.001, compared to the untreated group; (c). Dynamic effects of LINC01094 and its target miR-1266-5p on expression of *SLPI* mRNA. *P < 0.05, ***P < 0.001, compared to the si-NC+anti-miR-con group; ###*P* < 0.001, compared to the si-NC+anti-miR-1266-5p group.

TargetScan was used to predict the downstream targets of miR-1266-5p. *SLPI*, which has been reported as an upregulated and promoter in CRC [29,30], was identified as a potential candidate gene (Figure 6a). In a dual-luciferase reporter assay, miR-1266-5p mimics significantly inhibited the relative luciferase activity of ***SLPI***-WT, but not ***SLPI*** -MUT, while miR-1266-5p inhibitor played the opposite role (*P* < 0.01, Figure 6b). Moreover, the effects of LINC01094 siRNA and miR-1266-5p inhibitor on *SLPI* mRNA in CRC cells were then evaluated. Compared with negative control, *SLPI* expression was significantly decreased at the mRNA level in CRC cells transfected with LINC01094 siRNA but increased in CRC cells transfected with miR-1266-5p inhibitor (*P* < 0.05, Figure 6c). Besides, miR-1266-5p inhibitor partly recovered the expression of SLPI mRNA reduced by LINC01094 siRNA. Therefore, in CRC, LINC01094/miR-1266-5p may modulate cell biology through *SLPI*.

## Discussion

In this study, it was demonstrated that the LINC01094 was significantly increased in CRC tissues and cells. And its highly expression was correlated with poor prognosis and cancer progression of CRC patients. Finally, LINC01094 was identified as an independent prognostic biomarker for CRC. Through a series of cellular functional and mechanistic assays, LINC01094 was found to facilitate the CRC cell proliferation, migration, and invasion, which imply LINC01094 serves as an oncogene in CRC by competitively binding to miR-1266-5p and influencing the expression of *SLPI*.

CRC continues to be one of the health burdens worldwide owing to the delayed diagnosis and the poor prognosis due to recurrence or metastasis. Given the existing tools for the prognosis and therapy of CRC remain suboptimal, better strategies especially some effective prognostic prediction methods are required to replace or supplement the current tools. LncRNAs are involved in CRC development, such as lncRNA PANDAR, HOTAIR, LINC00052 [31–33]. As was revealed by previous studies, LINC01094 can facilitate the progression of ovarian cancer, glioma, and clear cell renal cell carcinoma [17,18,24]. In the present study, it is figured out that LINC01094 was upregulated in CRC tissues and cells, and strongly associated with malignant features of CRC, such as absent vascular invasion, positive lymph node metastasis, and advanced TNM stage. Further Kaplan-Meier analysis and multivariate analysis demonstrated LINC01094 was an effective and strong biomarker for CRC overall survival and progression-free survival. These also suggested that LINC01094 might involve in the cancer progression of CRC.

LncRNAs have been revealed as key regulators in a wide range of biological processes [34]. Cell proliferation, differentiation, and metastasis are the key cellular functions regulated by lncRNAs [35,36]. For instance, LncRNA DCST1-AS1 is significantly up-regulated in CRC tissues and cell lines, and its silence inhibits the proliferation, migration, and invasion of CRC cells [37]. Otherwise, overexpression of LINC00662 goosed the occurrence and development of colon cancer by moderating miR-340-5p. Likewise, LINC01094 was reported to promote the tumorigenesis and metastatic phenotypes of glioblastoma cells as a ‘sponge’ for miR-126-5p [38]. We here explored the role of LINC01094 in CRC cellular function and found inhibition of LINC01094 led to the suppression of cell growth, migration, and invasion. This suggests LINC01094 may function as an oncogene in CRC.

Ongoing studies continue to unravel the ceRNA hypothesis of lncRNAs in cancers. This lncRNA-miRNA communication likely contributes to the repressive effect of miRNA [39]. Finding the further experimental evidence of the lncRNA-miRNA interaction was the main way to clarify the involvement of lncRNA in the progression of cancers [40]. LINC01094 has been verified to promote the progression of breast cancer by sponging microRNA-340-5p and then regulating E2F transcription factor 3 [41]. In this study, miR-1266-5p was predicted as downstream miRNA of LINC01094 through bioinformatic analysis. Cell experiment, RNA pull-down assay, and dual-luciferase reporter assay verified this. Concerning the target genes of miR-1266-5p, *SLPI* is one of the verified oncogenes in CRC. *SLPI* mRNA level was significantly upregulated in CRC tissues compared to adjacent normal controls [29]. Moreover, *SLPI* silence inhibited proliferation, migration, and invasion of CRC cells and *SLPI* upregulation is associated with a poorer prognosis [30]. As So, we speculate LINC01094 promotes CRC cells’ malignant biological behavior at least mostly through regulating miR-1266-5p/*SLPI*.

However, our study had some limitations. First, the samples were limited to a single institution and the sample size was small. Second, the cancer-promoting effect of LINC01094 lacks validation in the animal in vivo experiments.

## Conclusion

This research revealed the upregulation of LINC01094 in CRC, which predicts poor overall survival and cancer progression. LINC01094 may expedite the growth, migration, and invasion of CRC cells by sponging miR-1266-5p. This study shed light on the clinical significance of LINC01094 as a prognostic predictor and a new therapeutic target.

## Supplementary Material

Supplemental Material

## Highlights

- Levels of LINC01094 increase in tissues of colorectal cancer patients and cells.
- LINC01094 level associated with unfavorable clinicopathological factors.
- LINC01094 predicted poor prognosis of colorectal cancer.
- Downregulation of LINC01094 impeded the cell malignant biological behavior.
