# Supplementary material for: Upregulated long intergenic non-protein coding RNA 1094 (LINC01094) is linked to poor prognosis and alteration of cell function in colorectal cancer
Source: Bioengineered. 2022 Mar 24;13(4):8526–37. doi: 10.1080/21655979.2022.2051839 (PMC9161846; doi:10.1080/21655979.2022.2051839)
Supplement: Supplemental Material [file KBIE_A_2051839_SM7336.docx]

Supplement Table 1. The primers sequences used in RT-qPCR were listed below.

| Gene | Forward Primer (5' - 3') | Forward Primer (5' - 3') |
| --- | --- | --- |
| LINC01094 | AGCCTCGGCTGTGTTTGTAT | AGGTTGACACATCTCGCCTG |
| SLPI | CCCTTCCTGGTGCTGCTT | CCTCCTTGTTGGGTTTGG |
| GAPDH | TCGGAGTCAACGGATTTGGT | TTCCCGTTCTCAGCCTTGAC |
| MiR-1266-5p | GCCGAGCCTCAGGGCTGTAGAAC | CTCAACTGGTGTCGTGGA |
| U6 | CTCGCTTCGGCAGCACA | AACGCTTCACGAATTTGCGT |
